# The Prediction of Students’ Academic Performance With Fluid Intelligence in Giving Special Consideration to the Contribution of Learning

**DOI:** 10.5709/acp-0175-z

**Published:** 2015-09-30

**Authors:** Xuezhu Ren, Karl Schweizer, Tengfei Wang, Fen Xu

**Affiliations:** 1 School of Education, Huazhong University of Science & Technology, Wuhan 430074, China; 2 State Key Laboratory of Cognitive Neuroscience and Learning, Beijing 100875, China; 3 Goethe University Frankfurt, Gruneburgplatz 1, 60323 Frankfurt a. M., Germany; 4 Department of Psychology, Zhejiang Sci-Tech University, Hangzhou 310018, China

**Keywords:** individual differences fluid intelligence, complex learning, academic performance

## Abstract

The present study provides a new account of how fluid intelligence influences academic performance. In this account a complex learning component of fluid intelligence tests is proposed to play a major role in predicting academic performance. A sample of 2, 277 secondary school students completed two reasoning tests that were assumed to represent fluid intelligence and standardized math and verbal tests assessing academic performance. The fluid intelligence data were decomposed into a learning component that was associated with the position effect of intelligence items and a constant component that was independent of the position effect. Results showed that the learning component contributed significantly more to the prediction of math and verbal performance than the constant component. The link from the learning component to math performance was especially strong. These results indicated that fluid intelligence, which has so far been considered as homogeneous, could be decomposed in such a way that the resulting components showed different properties and contributed differently to the prediction of academic performance. Furthermore, the results were in line with the expectation that learning was a predictor of performance in school.

## Introduction

Numerous studies have demonstrated that intelligence is a main predictor of academic performance (e.g., Deary, Strand, Smith, & Fernandes, 2007; Watkins, Lei, & Canivez, 2007). Fluid intelligence that has been found to be especially closely related to general intelligence (Kvist & Gustafsson, 2008; McArdle & Woodcock, 1998) has frequently played a leading role in studies on the relationship with academic performance. Although this relationship has been regarded as a well-established fact, the source of the relationship still seems to be in need of a convincing account. Cattell’s (1963, 1987) investment hypothesis stating that individuals invest their fluid intelligence to acquire strategies and knowledge can be considered as an attempt to provide an account. More recently, the research has shifted to focus on the underlying cognitive processes. Attempts have been made to understand why and how complex cognitive processes influence students’ academic performance (e.g., Ferrer & McArdle, 2004; Krumm, Ziegler, & Buehner, 2008). This paper adds another approach to this line of research: Fluid intelligence is decomposed into components showing different cognitive properties and contributing differently to the prediction of academic performance.

### The position effect observed in intelligence tests

The new approach originates from the position effect research. This effect has frequently been observed in items of intelligence tests. It denotes the dependency of responses to items on the position of the items within a test (Schweizer, Troche, & Rammsayer, 2011). Since intelligence tests are composed of a number of items showing a high degree of similarity, there is a high possibility of observing the position effect among the items within a test (e.g., Kubinger, Formann, & Farkas, 1991; Schweizer et al., 2011; Schweizer, Schreiner, & Gold, 2009). Further, a few empirical studies have suggested that learning serves as the source of the position effect in intelligence items (Embretson, 1991; Ren, Wang, Altmeyer, & Schweizer, 2014; Verguts & De Boeck, 2000). This position effect provides the outset to investigate the question whether the assumed learning processes underlying the position effect could account for the relationship between fluid intelligence and academic performance.

The research on the position effect has a long history starting in the 50s (Campbell & Mohr, 1950). The work by Knowles (1988) who observed that in personality scales item reliability increases as a function of the item serial position was especially enlightening. The position-related change was also found in ability tests such as the Raven’s Standard Progressive Matrices (Kubinger et al., 1991). The results of these studies indicate that the response to the items becomes increasingly consistent as testing continues. The more recent focus of this line of research is to represent the position effect observed in intelligence items by means of advanced confirmatory factor analysis (CFA) models (e.g., Ren, Goldhammer, Moosbrugger, & Schweizer, 2012; Schweizer et al., 2009). These CFA models decomposed the variance of intelligence test data into a position component that is associated with the position effect, and a constant component that is independent of the item positions. The research work by Schweizer et al. (2011) indicated that the constant component of fluid intelligence may represent basic cognitive processes and was highly correlated with general intelligence. However, the nature of the position component received little attention in this study.

### Complex learning as source of the position effect accounts for academic performance

There are reasonable grounds suggesting learning as the source of the position effect. First, the position effect appears to be associated with the similarity among the items of a test and the similarity provides opportunities for test-takers to detect the regularities and extrapolate them from one item to the next one. Since items of many fluid intelligence tests are dominated by only a few underlying rules (Carpenter, Just, & Shell, 1990), it is quite likely that test-takers are able to infer these rules and improve their ability to solve the items as testing continues. Second, previous research work conducted in the framework of IRT suggested that such kind of learning did occur in completing items of an intelligence test even without direct external feedback (e.g., Fischer & Formann, 1982; Verguts & De Boeck, 2000).

The nature of learning associated with the position effect of intelligence items was made explicit by a more recent study in considering both associative learning and complex learning (Ren et al., 2014). While associative learning represents an individual’s ability to form and maintain new associations between the knowledge items stored in memory, complex learning mainly reflects an individual’s ability to acquire and develop a series of goal-directed strategies based on the use of abstract rules (cf. Anderson, Fincham, & Douglass, 1997). The study by Ren et al. (2014) related the position and constant components of Raven’s Advanced Progressive Matrices (Raven’s APM), a well-known marker of fluid intelligence, to measures of associative learning and complex learning. Based on a sample of 220 university students the results of the study demonstrate that complex learning displays an especially strong link (*r* = .78) with the position component while associative learning shows only a small correlation (*r* = .28) with the constant component of Raven’s APM.

The revelation of complex learning as the main source of the position effect was especially revealing with respect to the prediction of academic outcomes on the basis of fluid intelligence. Fluid intelligence has been considered as a causal factor in learning activities, especially in novel situations (Kvist & Gustafsson, 2008). This argument has been bolstered by empirical studies demonstrating a substantial relationship between learning and fluid intelligence when the learning tasks are new and complex (e.g., Tamez, Myerson, & Hale, 2008). Additionally, the investment hypothesis and related empirical research suggest that fluid intelligence supports the acquisition of skills and knowledge across a wide spectrum of domains including arithmetic skills and vocabulary (Cattell, 1987; Ferrer & McArdle, 2004). Therefore, it appears reasonable to hypothesize complex learning as an underlying source that gives rise to the association between fluid intelligence and knowledge acquisitions.

### The aim of the present study

As elaborated in the previous section, it is possible to separate a learning component based on the position effect of intelligence items from a constant component by means of theory-based CFA models. The position-related component has been demonstrated to show a close relationship with measures of complex learning, indicating that complex learning is a major source of the position effect of intelligence items. The aim of the present study was therefore to examine the role of this learning component in accounting for academic performance. To that end, measures of fluid intelligence and academic performance were administrated to a large sample. Variance of the intelligence data was decomposed into the position and constant components by special CFA models. Since complex learning abilities have been indicated as the main source of the position effect observed in intelligence items, it was hypothesized that the position component of fluid intelligence played a key role in predicting academic performance.

## Method

### Participants

The data of the present study came from a large research project conducted across China to assess children’s and adolescents’ cognitive, academic and social development. The sample used for this paper was defined by students enrolled at 10 junior secondary schools located at a medium-sized city in south China. There were 2,277 students (1,176 males and 1,101 females) in the second year of the junior secondary schools with an average age of 13.53 years (*SD* = 0.28). Data were collected at the beginning of the academic year. Since the reasoning tests and the academic tests were administered separately (within one week), a total of 17 participants had missing scores on either the reasoning scores or the academic scores. The loss was very small because data collection was conducted during normal teaching time, and absence from school is rare in China. Data of those participants were excluded from analysis.

### Measures

The measures included two analogical reasoning tests (figural and numerical versions) to assess fluid intelligence. Academic performance was assessed by standardized math and verbal tests. All these tests came from the test reservoir developed for the national research project^1^ and have gone through rigorous construction processes (Dong & Lin, 2011).

#### Reasoning tests

**Figure 1.**
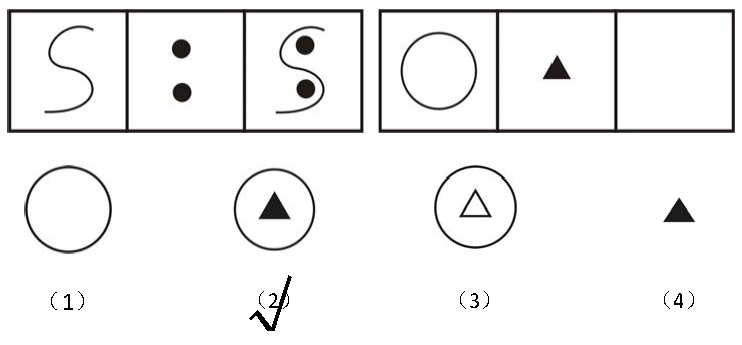
An example of the item of the figural reasoning test with the correct answer.

Fluid intelligence was assessed using analogy tasks combining different contents. The figural reasoning (FR) test consisted of 19 items each presented in the form of analogy patterns composed of geometric figures (see Figure 1 for an example). To complete each item, participants had to infer the rule underlying the first pattern and to apply the rule to complete the second pattern by choosing a correct figure out of four alternatives. The 19 items of this test were presented in an ascending order of difficulty. The numerical reasoning (NR) test was the numerical equivalent of the FR test. The elements of the patterns were simple numbers composed according to underlying rules. This test consisted of 22 items presented also in an ascending order of difficulty. Participants had 8 min to complete each test. The time limit was chosen on the basis of the results of several pilot testing sessions to make sure that participants had sufficient time to try to complete each item of each test. The response to each item of the tests was recorded as binary data. According to the technical report of these tests (Dong & Lin, 2011), internal consistency indexed by Cronbach’s s was computed based on a national norm of 12,000 junior middle school students. The internal consistencies were .77 for the FR and .86 for the NR. Criterion validity of the reasoning test was established on the basis of 120 students. The Matrix Reasoning subtest of the Wechsler Intelligence Scale for Children (WISC-IV) served as an external criterion for the reasoning test. Correlations of the FR and NR tests with WISC-IV Matrix Reasoning were .66 (*p* < .01) and .64 (*p* < .01) respectively.

#### Academic tests

The math and verbal tests were constructed strictly according to curriculum standards set by the state department of education for junior secondary education. The math test included 26 multiple-choice items and 6 open items. These items covered three dimensions of the math curriculum: algebra, geometry, and probability. The verbal test included 38 multiple-choice items covering two major dimensions of the verbal curriculum: comprehension and literacy knowledge. Participants had 60 min to complete each test. Separate scores were calculated for each dimension of the tests. According to the technical report of the tests (Dong & Lin, 2011), the internal consistencies of the math and verbal tests were .88 and .80 respectively. Convergent validity of the tests was assessed by computing the correlations of the dimension scores with the total test scores. Correlations of the algebra, geometry, and probability with the total math score were .94, .93, and .64 respectively. Correlations of the comprehension and literacy knowledge with the total verbal score were .94 and .92 respectively.

It should be noted that there were three parallel versions of each academic test, and that these tests shared a set of common items known as anchor items. The equation of the scores obtained from the three parallel versions was achieved by means of the one parameter logistic model (for the multiple-choice items) and the partial credit IRT model (for the open items). These scores were used for representing academic achievement.

### Statistical analysis

Individual items provided the basis for analyzing the data of the reasoning tests. The research approach selected for decomposing and representing the constant and the position components of the reasoning tests were special CFA models addressed as the fixed-links models (cf. Schweizer, 2008). A characteristic of the fixed-links models is that factor loadings are constrained according to theory-based expectation so that the variances of the manifest variables are decomposed into independent components. Independence of the latent components means that latent variables are prevented from accounting for the same variances and covariances. If the latent variables were allowed to correlate with each other, this would very likely lead to substantial correlations of both latent variables with the same criterion measures. In this case, it may become virtually impossible to demonstrate whether the increasing component that represents the position effect is correlated to a higher degree with the criterion than the other latent variable.

**Figure 2.**
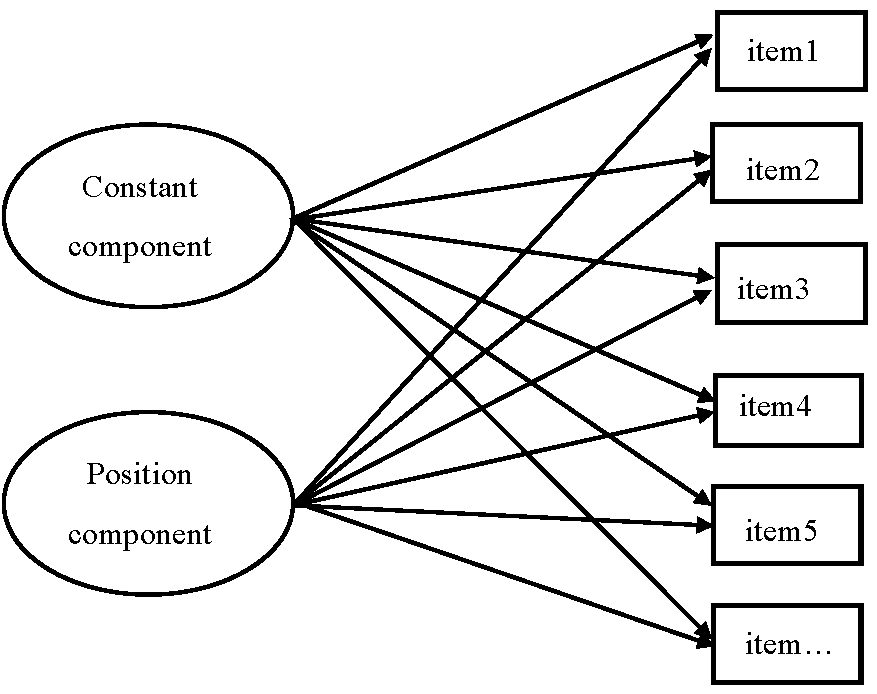
An illustration of the measurement model including the constant and position components of reasoning as two independent latent variables and the individual items of each reasoning test as manifest variables (the model of the figure reasoning includes 19 manifest variables, and the model of the numerical reasoning includes 22 manifest variables).

The representation of the position effect for each reasoning test required a fixed-links model including two latent variables: the constant component and the position component. Figure 2 illustrates the measurement model including the constant and position components of reasoning and the individual items of each reasoning test serving as manifest variables. The loadings of the constant component were kept constant since this component was independent of item positions and contributed almost equally to all individual items. The loadings of the position component were determined by a quadratic function (e.g., 1, 4, 9…) that described the influence of complex learning on the position effect—that is, a small increase may occur at the first few positions whereas a steep slope is achieved as one progresses through the test. A simple linear function was also considered to represent the position effect for a comparison. This linear function simply means that learning increases linearly as testing continues from the first to last items. These two fixed-links models were addressed as Linear- and Quadratic models. Since there was the necessity to relate the binomial distributions of the binary reasoning items to the normal distributions of the latent scores, a link transformation for eliminating effects due to such a discrepancy was adopted (cf. McCullagh & Nelder, 1985). This transformation was accomplished by weights serving as multiplier to each true component of the measurement models.

A single factor model that did not consider the position effect was also investigated. This model was address as the constant model. The loadings of the latent variable were kept the same as those of the constant component in the linear and quadratic models. Table 1 presents the fixed loadings that were inserted into each measurement model.

**Table 1.** The Fixed Loadings of Each Manifest Variable on the Constant Component and the Position Component of the Measurement Models

Number of item	Figural reasoning test					Numerical reasoning test				
	*M*	*SD*	Constant	Position (Q)	Position(L)	*M*	*SD*	Constant	Position(Q)	Position(L)
1	.99	0,06	.0628	0,0628	0,0628	.99	0,11	.1083	0,1083	0,1083
2	.98	0,10	.1042	0,4169	0,2085	.98	0,13	.1265	0,5058	0,2529
3	.98	0,14	.1392	1,2529	0,4176	.99	0,11	.1063	0,9564	0,3188
4	.96	0,18	.1842	2,9465	0,7366	.95	0,21	.2098	3,3564	0,8391
5	.92	0,28	.2759	6,8987	1,3797	.99	0,11	.1102	2,7558	0,5512
6	.80	0,40	.3976	14,3147	2,3858	.98	0,13	.1330	4,7881	0,7980
7	.90	0,30	.2967	14,5382	2,0769	.98	0,13	.1265	6,1966	0,8852
8	.86	0,35	.3449	22,0718	2,7589	.96	0,18	.1842	11,7862	1,4733
9	.68	0,47	.4676	37,8794	4,2088	.94	0,24	.2442	19,7831	2,1981
10	.73	0,44	.4427	44,2730	4,4273	.95	0,21	.2126	21,2605	2,1261
11	.85	0,36	.3569	43,1865	3,9260	.97	0,17	.1785	20,3041	1,8458
12	.70	0,46	.4564	65,7211	5,4768	.97	0,18	.1678	25,7060	2,1422
13	.85	0,36	.3569	60,3183	4,6399	.97	0,17	.1666	28,1494	2,1653
14	.63	0,48	.4824	94,5455	6,7533	.91	0,29	.2850	55,8651	3,9904
15	.74	0,44	.4402	99,0355	6,6024	.78	0,42	.4167	93,7594	6,2506
16	.59	0,49	.4912	125,7568	7,8598	.71	0,45	.4530	115,9260	7,2476
17	.59	0,49	.4917	142,0874	8,3580	.74	0,44	.4361	126,0176	7,4128
18	.57	0,50	.4948	160,3144	8,9064	.83	0,38	.3768	122,0944	6,7830
19	.55	0,50	.4974	179,5590	9,4505	.59	0,49	.4930	177,7179	9,3536
20						.61	0,49	.4880	195,1812	9,7591
21						.46	0,50	.4982	219,7045	10,4621
22						.68	0,47	.4660	225,5239	10,2511

*Note*. The loadings on the position component were determined by either a quadratic (Q) or a linear (L) function combined with the link transformation.

The statistical investigations were conducted by means of LISREL 8.8 (Jöreskog & Sörbom, 2006) on the basis of the covariance matrix, and model parameters were estimated by means of the maximum likelihood method. The fit statistics 2, Root Mean Square Error of Approximation (RMSEA), The Standardized Root Mean Square Residual (SRMR), and Confirmatory Fit Index (CFI) were considered. The limits proposed by Kline (2005) were referenced to evaluate the model-data fit. In addition, competing non-nested models were compared on the basis of Akaike Information Criterion (AIC). Lower AIC values reflect better model-data fit, and the model with the lowest AIC value is preferred.

## Results

The item-based scores of the reasoning tests are presented in Table 1. Descriptive results for the two reasoning tests, the math and verbal tests and their respective dimensions, as well as the intercorrelations among the variables are presented in Table 2. All correlations reached significance at the .01 level (two-tailed).

**Table 2.** Descriptive Statistics For the Two Reasoning Tests, the Math and Verbal Tests, and Their Respective Dimensions, as Well as the Intercorrelations Between the Variables (*N* = 2,277)

Measure	*M*	*SD*	1.	2.	3	4.	5.	6	7.	8.
1. Figural reasoning test	13,91	2,61	–							
2. Numerical reasoning test	18,93	2,64	.55	–						
3. Math	0,95	0,89	.55	.59	–					
4. Algebra	0,73	0,25	.49	.55	.91	–				
5. Geometry	0,72	0,30	.51	.51	.90	.68	–			
6. Probability	0,63	0,30	.27	.27	.52	.38	.38	–		
7. Verbal	1,11	0,63	.45	.50	.66	.59	.58	.38	–	
8. Literacy knowledge	0,72	0,16	.41	.46	.59	.55	.50	.34	.86	–
9. Comprehension	0,68	0,16	.35	.39	.53	.43	.49	.35	.83	.50

*Note*. The scores of the reasoning tests are the averaged total number of the correctly completed items; the scores of the academic tests are IRT-based scores.

### The representation of the components of fluid intelligence

As described in the Method section, three measurement models were examined for each reasoning test. Table 3 presents the fit results of the models. A comparison of the constant model and the other two models for each reasoning test clearly indicated that the consideration of the position effect reduced the 2 and AICs considerably. Although the outcomes of CFIs for the position-related models were not very favorable, they could be considered as acceptable since the large sample size affected the statistics on which the CFI was based. Table 3 also indicates that the quadratic models showed better fits than the linear models, as can be seen from the obviously lower AIC value of the quadratic models. These fit results indicate an advantage of representing the position effect according to the quadratic function. Therefore, the two quadratic models were selected for further analyses. The scaled variances (cf. Schweizer, 2011) of the latent variables within each of the selected models reached the level of significance, constant of FR: σ = .0116, *t* = 18.62, *p* < .01, position of FR: σ = .0045, *t* = 6.61, *p* < .01; constant of NR: σ = .0136, *t* = 23.80, *p* < .01, position of NR: σ = .0121, *t* = 15.76, *p* < .01. It should be noted that these statistical results were generated by the LISREL program.

**Table 3.** Fit Statistics of the Measurement Models for Each Reasoning Test.

Type of model	χ^2^	*df*	RMSEA (CI90)	SRMR	CFI	AIC
Figural reasoning test						
Constant	960,56	170	.045(.042..048)	.048	.819	1000,56
Linear	1062,85	169	.048 (.045..051)	.060	.504	1104,85
Quadratic	856,34	169	.042 (.039 ..045)	.048	.835	898,34
Numerical reasoning test						
Constant	3895,73	230	.084 (.081 ..086)	.087	.079	3941,73
Linear	3302,60	229	.077 (.074 ..079)	.089	.811	3350,60
Quadratic	2929,86	229	.072 (.070 ..074)	.092	.824	2977,86

*Note*. RMSEA = Root Mean Square Error of Approximation, SRMR = Standardized Root Mean Square Residual, CFI = Confirmatory Fit Index, AIC = Akaike Information Criterion.

Next, a comprehensive CFA model that allowed the two constant components and the two position components of the reasoning tests to correlate with each other was inspected. This model showed an overall acceptable fit, χ^2^(812) = 4,598.64, RMSEA = .045 [CI90: .044.047], SRMR = .067, CFI = .865. Table 4 provides the latent correlations among the four components. As expected, substantial correlations were observed between the two position components and between the two constant components. The other correlations between the latent components were at only a weak or moderate level of significance.

**Table 4.** Completely Standardized Correlations Between the Latent Components of the Two Reasoning Tests

Latent component	Figural reasoning test	
Constant	Position
Numerical reasoning test		
Constant	.51**	.37*
Position	.12	.65**

**Figure 3.**
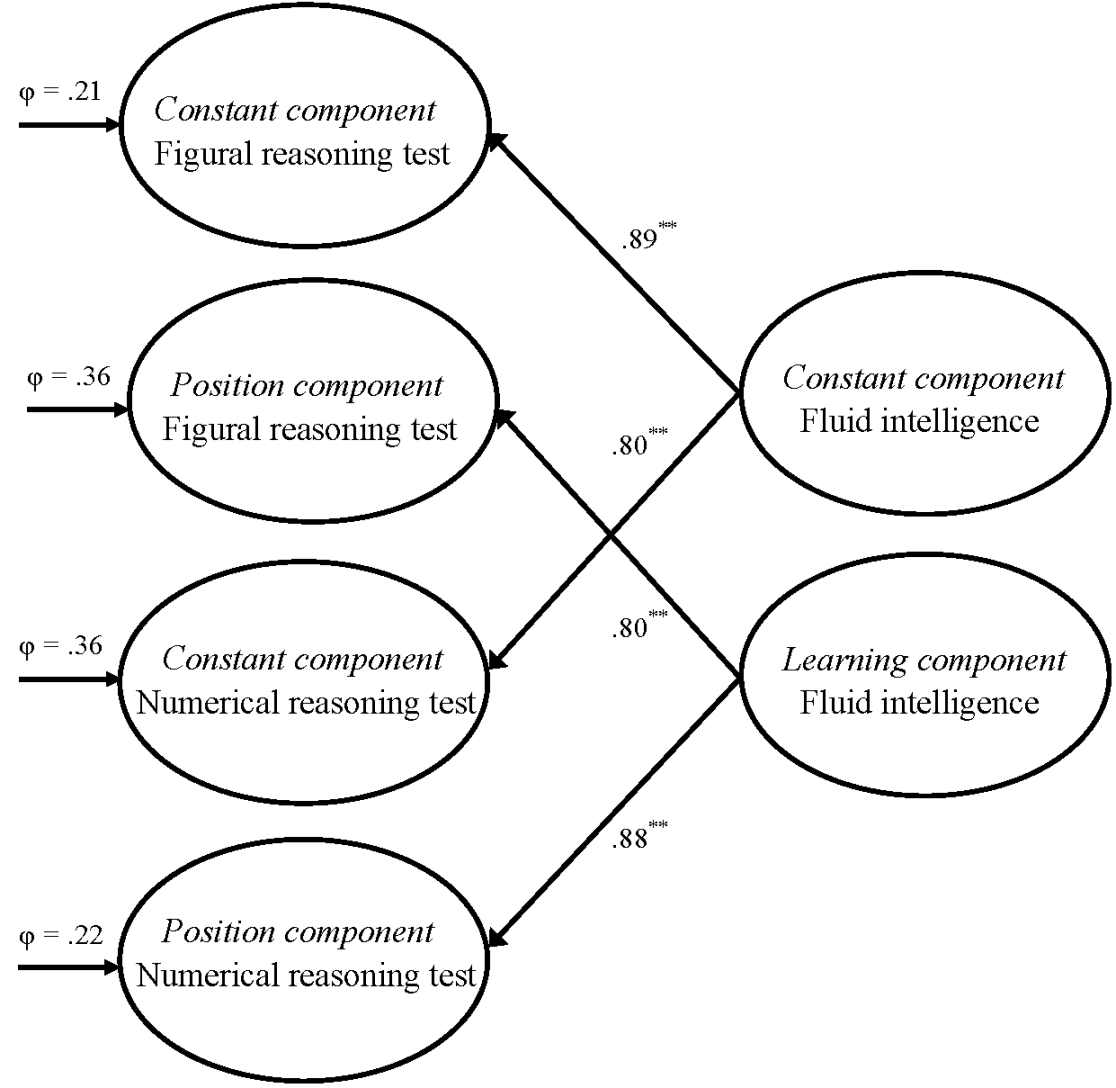
The latent structure of the second-order CFA model with the constant and learning components of fluid intelligence as higher-order factors which were derived from the four components of the reasoning tests. Completely standardized factor loadings and completely standardized error variances of the latent variables are also presented (** *p* < .01). The correlations between the constant and the position components were fixed to zero.

In a following step, a second-order CFA model that included two higher-order factors representing the constant and the learning components of fluid intelligence was inspected. This second-order model, compared to the comprehensive CFA model, additionally included two higher-order factors addressed as the constant and learning components of fluid intelligence. Figure 3 presents the latent structure of this second-order model. Unfortunately, some of the estimated parameters could not be identified in this model. Therefore, we fixed the residuals of the first-order latent variables according to the estimated values from the comprehensive CFA model (i.e., the first-order model) so that a stable switch was achieved from the first- to the second-order models. The fit statistics of the second-order model were acceptable, χ^2^(816) = 4,743.26, RMSEA = .046 [CI90: .045 .047], SRMR = .065, CFI = .862. The relationships of the first-order latent variables and the second-order latent variables were rather close, as it was obvious from the standardized loadings varying between .80 and .89.

### Accounting for academic performance by components of fluid intelligence

**Figure 4.**
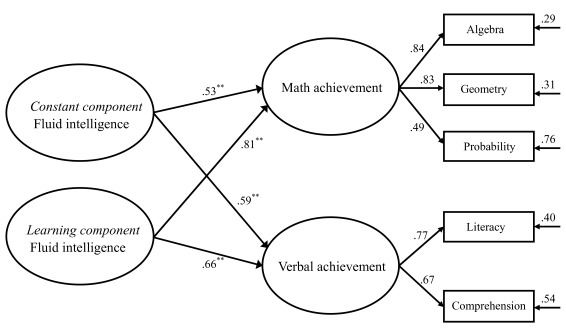
The prediction model including the constant and learning components of fluid intelligence as predictor variables and math and verbal achievements as predicted variables. All completely standardized path coefficients reached the level of significance (** *p* < .01). The path coefficient from the learning component to each predicted variable was statistically larger than the one from the constant component.

The representation of the constant and learning components of fluid intelligence by the second-order CFA model made it possible to relate the components to the academic scores. This was achieved by a means of a full structural equation model additionally including two criterion variables representing the math and verbal performance. The fit statistics of this model indicate a good fit, χ^2^(1018) = 5, 204.97, RMSEA = .043 [CI90: .041 .044], SRMR = .063, CFI = .915. Figure 4 provides an illustration of the structure of this prediction model.

Overall, moderate to strong relationships were found between the components of fluid intelligence and the latent variables of academic performance. A surprisingly strong link was observed from the learning component to math performance. This link was stronger than the one from the constant component of fluid intelligence to math performance, *Z*_difference_ = 18.10, *p* < .01. A further analysis of the two path coefficients suggested that the learning component accounted for 66% of the latent variance of math performance and the constant component accounted for 28%. With respect to predicting verbal performance, the corresponding coefficients indicated that the learning component played a slightly more important part than the constant component, *Z*_difference_ = 3.88, *p* < .01. Further inspection of the two path coefficients revealed that the learning component accounted for 44% of the latent variance of verbal performance and the constant component accounted for 35%. In addition, the residual correlation between verbal and math performance was only .05, indicating that they were not associated with each other after the variance due to fluid intelligence was removed.

## Discussion

So far, there has been hardly any empirical evidence regarding the assumption that learning capacity incorporated in conceptualizations of intelligence contributes to students’ academic performance. The present study attempted to provide this evidence. The perspective of the position effect suggests that the learning component of fluid intelligence may play a crucial role in predicting academic performance. The fixed-links modeling approach was employed to separate the learning component of fluid intelligence from a constant component. The two components of fluid intelligence were linked to math and verbal performance. The results showed that the complex learning component played a more important part than the other component of fluid intelligence in predicting math and verbal performance. The link from the learning component to math performance was especially obvious. These results suggest that the reason why fluid intelligence predicts academic outcomes is that highly intelligent individuals are especially efficient in learning new skills in novel and complex situations, which seems to lead to high potential for achieving success in academic activities.

The present finding was in accordance with, and updated two lines of previous research. One line of research conducted in the framework of psychometric studies has found a positive relationship between fluid intelligence and the rate of learning, or learning in real-life situations (e.g., Klauer & Phye, 2008; Tamez et al., 2008). This line of research suggests that a fundamental aspect of fluid intelligence is the ability to learn in novel situations, as was clearly demonstrated by the current study that a learning component was represented and derived from measures of fluid intelligence. Furthermore, the findings of the current study updated previous work that was conducted to test the investment hypothesis which provides insight into the learning function of fluid intelligence for acquiring strategies and knowledge (Ferrer & McArdle, 2004). Although direct evidence supporting Cattell’s (1963, 1987) investment hypothesis was limited by the cross-sectional nature of this study, the result that the learning component of fluid intelligence had a substantial correlation with math and verbal performance underscored the importance of the learning function implicated in fluid intelligence.

It is necessary to note that since the learning and the constant components of fluid intelligence were not orthogonal, it was quite likely that these two components accounted for an overlapping part of the variance of math or verbal performance. In spite of that, it was clear from the current result that the learning component played a more important part than the other component of fluid intelligence in predicting academic performance. In addition, although those components of fluid intelligence accounted for a large part of the variances of academic performance, other factors such as conscientiousness, motivation, and so forth should also play a crucial role in predicting students’ academic achievements (e.g., Mega, Ronconi, & De Beni, 2014). Lastly, concerning the fit statistics of the measurement models, although both RMSEAs and SRMRs were acceptable, the CFIs were not at or above .90. This finding may partly be due to the large number of variables within each model (cf. Kenny & McCoach, 2003).

To conclude, the current study decomposed measurements obtained by two reasoning measures into two components and showed that these components differently related to two types of academic achievement. The results indicate that reasoning data, which have been considered as homogeneous, can be decomposed in such a way that the resulting components show different properties. Furthermore, the results are in line with the expectation that learning is a predictor of performance in school. To be more specific, the position component that mainly reflects complex learning accounted for a larger part of the variance of academic performance than that of the constant component of fluid intelligence. These findings provide evidence of how tests of fluid intelligence predict academic performance and justify the use of intelligence tests as educational tools. Furthermore, the finding that the learning component of fluid intelligence predicts a substantial part of the variance of academic achievement provides empirical evidence supporting Cattell’s (1963, 1987) investment hypothesis, and also provides insight into the learning function of fluid intelligence for acquiring strategies and knowledge of various domains.

## Footnotes

1 This research project was China’s first attempt to assess children’s mental development and academic skills cooperatively accomplished by psychologists and educationists from 40 universities and institutes across China from year 2009 to year 2012.
